# Seed Dormancy and Soil Seed Bank of the Two Alpine *Primula* Species in the Hengduan Mountains of Southwest China

**DOI:** 10.3389/fpls.2021.582536

**Published:** 2021-04-14

**Authors:** De-Li Peng, Li-E Yang, Juan Yang, Zhi-Min Li

**Affiliations:** ^1^School of Life Sciences, Yunnan Normal University, Kunming, China; ^2^Faculty of Geography, Yunnan Normal University, Kunming, China; ^3^National Germplasm Bank of Wild Species, Kunming Institute of Botany, Chinese Academy of Sciences, Kunming, China

**Keywords:** alpine plant, base temperature, cold stratification, dry storage, light requirement, alternating temperature

## Abstract

The timing of germination has long been recognized as a key seedling survival strategy for plants in highly variable alpine environments. Seed dormancy and germination mechanisms are important factors that determining the timing of germination. To gain an understanding of how these mechanisms help to synchronize the germination event to the beginning of the growing season in two of the most popular *Primula* species (*P. secundiflora* and *P. sikkimensis*) in the Hengduan Mountains, Southwest China, we explored their seed dormancy and germination characteristics in the laboratory and their soil seed bank type in the field. Germination was first tested using fresh seeds at two alternating temperatures (15/5 and 25/15°C) and five constant temperatures (5, 10, 15, 20, and 25°C) in light and dark, and again after dry after-ripening at room temperature for 6 months. Germination tests were also conducted at a range of temperatures (5–30, 25/15, and 15/5°C) in light and dark for seeds dry cold stored at 4°C for 4 years, after which they were incubated under the above-mentioned incubation conditions after different periods (4 and 8 weeks) of cold stratification. Base temperatures (*T*_b_) and thermal times for 50% germination (*θ*_50_) were calculated. Seeds were buried at the collection site to test persistence in the soil for 5 years. Dry storage improved germination significantly, as compared with fresh seeds, suggesting after-ripening released physiological dormancy (PD); however, it was not sufficient to break dormancy. Cold stratification released PD completely after dry storage, increasing final germination, and widening the temperature range from medium to both high and low; moreover, the *T*_b_ and *θ*_50_ for germination decreased. Fresh seeds had a light requirement for germination, facilitating formation of a persistent soil seed bank. Although the requirement reduced during treatments for dormancy release or at lower alternating temperatures (15/5°C), a high proportion of viable seeds did not germinate even after 5 years of burial, showing that the seeds of these two species could cycle back to dormancy if the conditions were unfavorable during spring. In this study, fresh seeds of the two *Primula* species exhibited type 3 non-deep physiological dormancy and required light for germination. After dormancy release, they had a low thermal requirement for germination control, as well as rapid seed germination in spring and at/near the soil surface from the soil seed bank. Such dormancy and germination mechanisms reflect a germination strategy of these two *Primula* species, adapted to the same alpine environments.

## Introduction

The timing of germination is closely connected to the probability of seedling survival and thus seedling establishment (Jaganathan et al., 2015). This correlation is especially true in alpine regions, where seedlings must attain a critical size by the end of the short growing period to be able to survive the long, harsh winter (Wang et al., 2017). Seed dormancy is a main mechanism controlling the timing of germination in alpine plants (Schwienbacher et al., 2011; Jaganathan et al., 2015; Wang et al., 2017), preventing germination in autumn, and allowing seeds to delay germination until the spring when temperature, light, and water conditions are optimal for growth and survival of the seedlings. Baskin and Baskin (2014) have proposed a comprehensive dormancy classification system, which includes five classes: (1) physiological dormancy (PD), (2) morphological dormancy (MD), (3) morphophysiological dormancy (MPD), (4) physical dormancy (PY), and (5) a combinational dormancy (PY + PD). Of the arctic-alpine plants, more than 70% produce dormant seeds, exhibiting mainly PD, which evolved to prevent seed germination before and/or during cold autumn/winter seasons (Baskin and Baskin, 2014).

Three levels of dormancy are distinguished within PD (non-deep, intermediate, and deep), and non-deep is by far the most common (Baskin and Baskin, 2014). An important characteristic of seeds with non-deep PD is that dormancy release can occur during dry storage at room temperature; this phenomenon is called as after-ripening (Baskin and Baskin, 2020). Release from non-deep PD may also occur during dry storage at low temperatures (Wang et al., 2010). However, dormancy release may not be complete during dry storage, i.e., some seeds may become non-dormant while others remain dormant (Wang et al., 2010). In this case, cold stratification is regarded as an important way to release dormancy when dry storage cannot release dormancy completely, especially in seeds of most alpine plants (Schwienbacher et al., 2011; Rayburn et al., 2013; Cavieres and Sierra-Almeida, 2018; Yang et al., 2020). After dormancy release, the high temperature requirement for germination is reduced, and the range of temperatures which seeds will germinate, as well as germination rate and percentages, increase (Porceddu et al., 2013; Baskin and Baskin, 2014).

In addition to being dormant and having a temperature requirement, seed responses to light can also control the timing of germination from the soil seed bank (Jaganathan et al., 2015; Wang et al., 2017). A light requirement may prevent germination in deep soil and drives species to form persistent soil seed banks (Milberg et al., 2000). Some plants require light for germination even after the dormancy is broken (Brändel and Schütz, 2005; Jaganathan et al., 2015). Small seeds are easily buried deep in the soil, but light can penetrate only a few millimeters, so seeds requiring light for germination are usually small in size (Koutsovoulou et al., 2014). Milberg et al. (2000) suggested that a light response and seed mass coevolved as an adaptation to ensure germination of small-seeded species only when close to the soil surface. In the field, maximum daily temperature fluctuation usually occurs at or near the soil surface, especially in alpine regions. Therefore, the requirement for light and temperature fluctuations in some alpine species not only prevents deep soil germination in late autumn but also ensures germination occurs only at or near the soil surface during the next spring or the appropriate growing season (Jaganathan et al., 2015).

*Primula* L. is a large genus of approximately 430 species with the Hengduan Mountains and eastern Himalayas region supporting the greatest number of species (Yang, 2015). The seeds of many *Primula* species possess a type 3 non-deep PD and have relatively small size, requiring light for germination; these seed characteristics can easily lead to form a persistent soil seed bank (Milberg, 1994; Hitchmough et al., 2011). However, previous studies have usually focused on *Primula* species from the low-elevation woodlands or grasslands (Thompson, 1969; Hitchmough et al., 2011). Little is known of whether similar dormancy and soil seed bank types are found in other species, for example, the species from the high-elevation mountainous regions in the Hengduan Mountains.

In this study, we aimed to investigate the dormancy and soil seed bank types in *P. secundiflora* and *P. sikkimensis*, two popular primrose species in the high-elevation regions in the Hengduan Mountains. Seed dormancy and germination characteristics were affected by the interactions of seed traits, environmental factors, and phylogeny (Baskin and Baskin, 2014; Wang et al., 2016). Therefore, we hypothesized that (1) seeds of species from the high-elevation regions with low temperatures would have special thermal requirement for germination to adapt to this habitat and (2) the response of dormancy and germination to environmental factors are constrained by phylogeny, that is, species within the same genus from different habitats might have similar dormancy type and light requirement for germination. To test these hypotheses, we specifically addressed the following questions: (1) what is the dormancy type? How do dry after-ripening, dry cold-storage, and cold-wet stratification release dormancy and promote germination? (2) How do temperatures affect dormancy and germination? What are the cardinal temperatures and thermal time for germination after dormancy resulting in complete release? (3) How do light conditions affect germination in different states of dormancy? and (4) What are the soil seed bank types of the two species?

## Materials and Methods

### Species and Seed Collection

*Primula secundiflora* and *P. sikkimensis* grow in moist alpine meadows or on the borders of streams at altitudes between 3,200 and 5,100 m. Fresh seeds of *P. secundiflora* and *P. sikkimensis* were collected from at least 10 plants during September in three consecutive seasons (2013–2015; Table 1). The low number of sampled plants was due to the small number of fruiting individuals found in these two special habitats. After collection, except when fresh seeds were used directly in the experiments, seeds were allowed to after-ripen in a paper bag at room temperature for 6 months, and then stored in a sealed bag with silica gel below 4°C until the start of the experiments. When seeds had been air-dried for 3 months, the weight of 100 seeds was measured (Table 1).

**Table 1:** Collection sites of seeds for two *Primula* species.

Species	Region	Locality	Location	Altitude (m a.s.l.)	Habitat	Year	100 seed weights (mg)
*Primula secundiflora*	Yunan Province	HuLuHai Lake	99°57'24.33''E; 28°30'48.31''N	4,542	Alongside the stream in alpine meadow	2013	18.3 ± 0.55 (2013, *n* = 10)
						2014	18.8 ± 0.44 (2014, *n* = 10)
						2015	22.6 ± 0.14 (2015, *n* = 10)
*Primula sikkimensis*	Yunan Province	PuYong Pass	99°55'52.28''E; 28°24'49.37''N	4,550	Meadow patches in alpine scree	2013	71.2 ± 0.77 (2013, *n* = 10)
						2014	90.0 ± 3.80 (2014, *n* = 2)
						2015	65.9 ± 0.62 (2015, *n* = 9)

### Germination Tests of Fresh Seeds

In order to determine whether fresh seeds have PD, tests on such seeds were initiated 1 week after collection (using seeds collected in 2014 and 2015), on 1% water agar substrate, in plastic Petri dishes of 9 cm diameter in light under two alternating temperature regimes (15/5 and 25/15°C) in 2014 and five constant temperatures (5, 10, 15, 20, and 25°C) in 2015. To determine the effect of dark conditions, Petri dishes with seeds were wrapped in two layers of aluminum foil (in dark, hereafter), and then incubated at 5, 15, and 25°C. The daily photoperiod was 12 h light (22.2 μmol m^−2^ s^−1^ illumination from cool white fluorescent light), coinciding with the higher temperature phase, and 12 h dark (hereafter referred to as in light). At the study site, in the germination season (late April to late August), the daily temperature range is about 5–25°C near the soil surface. Thus, the alternating temperatures 25/15 and 15/5°C were selected to approximate daily temperature regimes during the germination season.

Due to limited availability of well-developed seed, three replicates of 20 seeds were used in each germination tests except in seed burial experiments. The Petri dishes were put into transparent plastic bags to prevent desiccation. Seeds incubated in light were counted every 3–4 days in germination tests of fresh seeds and counted every day in other tests. The criterion for germination was visible radicle protrusion and germinated seeds were discarded. To avoid exposure to light, the germination of dark-incubated seeds was checked only once at the end of the dark period. All experiments were terminated after 5 weeks, and then the viability of ungerminated seeds was checked by a cut-test. Seeds with a plump, firm, and white embryo were considered viable.

### Germination Tests After Dry Storage

Except some seeds that were used immediately in the experiments in 2014 and 2015, all fresh seeds were allowed to after-ripen in a paper bag at room temperature (25–55% relative humidity; 13–22°C). After 6 months of dry after-ripening (using seeds collected in 2014 and 2015), dried seeds were then moved to germination conditions, hereafter described as germination of fresh seeds. Due to limited seed availability in 2014 and 2015, the seeds used to test the effect of dry cold storage on germination were collected in 2013. After-ripening seeds incubated in light and dark under a range of constant temperatures (5, 10, 15, 20, 25, and 30°C) and alternating temperatures regimes (15/5 and 25/15°C) in June 2014, and after dry cold storage (<10% relative humidity; 4°C) for 4 years, seeds were then moved to germination conditions on August 2018, as described above.

### Germination Tests After Cold-Wet Stratification

After seeds had been in dry cold storage at 4°C for 4 years, two different cold-wet stratification (cold stratification, hereafter) periods were started on August 2018 (1°C in light in water 1% agar in 90 mm diameter plastic Petri dishes for 4 and 8 weeks: C1 and C2 treatments, respectively). Preliminary experiments showed that these temperature and light conditions were an efficient treatment to break dormancy in these two species. At the end of each pre-treatment, seeds were incubated in light under a range of temperatures (5, 10, 15, 20, 25, 30, 15/5, and 25/15°C). To determine the degree of the change in light requirements for germination under fluctuation temperatures after dormancy release, we also exposed some seeds to dark conditions under two alternating temperatures regimes (15/5 and 25/15°C), and two corresponding temperature (10 and 20°C) was selected as control treatment of the two alternating temperatures. Many of the seeds germinated during cold stratification at 8 weeks, and this germination was added to the final count in the light treatment, but not in the dark treatment.

### Seed Burial Experiments

In order to determine the type of soil seed bank, after 1 month of dry storage at room temperature (using seeds collected in 2014), samples of 30 plump seeds per species were put into bags (5 cm × 5 cm) made of polyester mesh. Nine seed bags were randomly buried on November 4, 2014 at a depth of 6 cm (based on field experience) in three places within a flat area at the collection site. The seed bags were covered with mulch without any changes in burial depth. Three bags were exhumed on October 24, 2015 (1-year burial), October 18, 2016 (2-year burial), and October 4, 2019 (5-year burial), respectively, and taken back to the laboratory. The contents of each bag were checked for seeds that had germinated (empty seed coat) or died (soft seeds) while buried. The viability of ungerminated and intact seeds was tested with germination experiments as described above at 20°C in light. After the germination experiment, ungerminated seeds were stained in 1% solution of 2,3,5-triphenyltetrazolium chloride for 24 h at 30°C. Seeds showing a strong red-stained embryo were considered viable.

### Data Analysis

The final germination percentage was expressed as the mean ± standard error (SE) of the three replicates according to the total number of filled seeds. Generalized linear models (GLMs) were used to compare: (1) whether dry after-ripening had significantly increased seed germination, relative to fresh seeds; (2) final germination percentages under different dry cold storage periods at a range of constant temperatures in both light and dark; (3) final germination percentages under different cold stratification periods at a range of constant temperatures in light; and (4) alternating and corresponding constant temperatures on germination (15/5 vs. 10 and 25/15 vs. 20°C) under different cold stratification periods in both light and dark. A *post hoc* pairwise comparisons t-test (with Bonferroni adjustment) was used to compare mean values of response variables as related to treatments.

Times to germination for non-dormant seeds can be modeled using a thermal-time approach (e.g., Porceddu et al., 2013; Zhang et al., 2020), in which a threshold cumulative quantity of thermal time (*θ*, °C days or h) is required above the base temperature (*T*_b_) to initiate germination. In this study, thermal time analyses were carried out for cold-stratified seeds germinating at constant temperatures. Because many seeds germinated during cold stratification for 8 weeks, this germination rate could not be calculated accurately, so we only calculated *θ*_50_ and *T*_b_ for seeds cold-stratified for 4 weeks. Germination time courses for all three replicates at a given temperature were combined and fitted using the Weibull Function (Brown and Mayer, 1988). Germination rate (GR, 1/*t*_50_), calculated as the inverse of the number of days (d^−1^) to reach 50% germination obtained from the cumulative germination progress curves, was plotted as a function of temperature and regressed using a linear model, to estimate the base temperature (*T*_b_) below which germination rate was equal to zero. The slope of the linear regression line corresponded to the reciprocal of the thermal-time requirement at suboptimal temperatures (*θ*_50_, °Cd). All data were analyzed using the procedures in PASW Statistics 18 (SPSS, Inc., 2009, Chicago, IL, www.spss.com), and all figures were drawn with OriginPro 9.5.

## Results

### Germination Tests of Fresh and Dry After-Ripening Seeds

**Figure 1:**
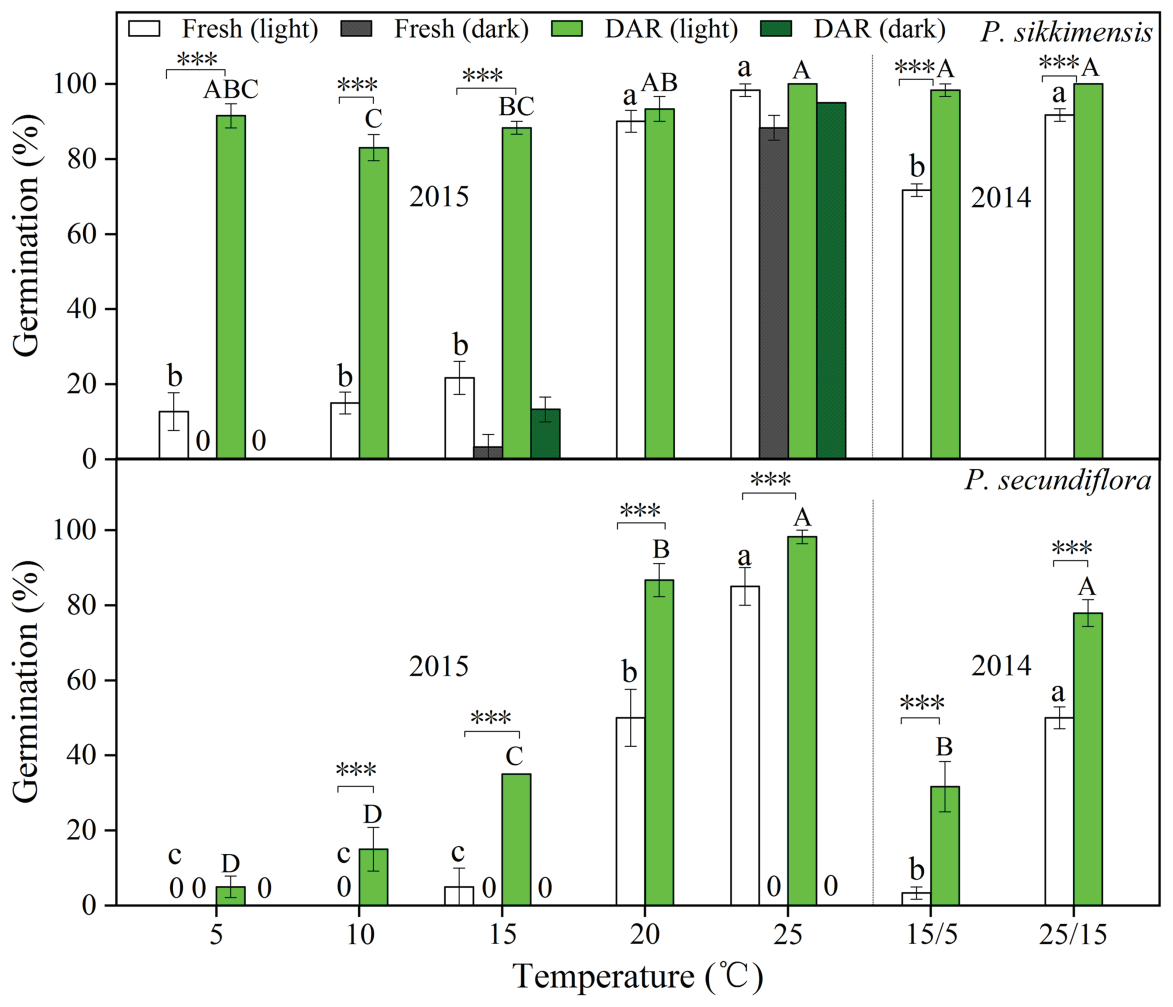
Final germination percentages in light and dark at two alternating temperatures (15/5 and 25/15°C) in 2014, and five constant temperatures (5, 10, 15, 20, and 25°C) in 2015 for fresh and dry after-ripening (DAR) seeds of *Primula secundiflora* and *Primula sikkimensis*. Error bars indicate SE for three replicates of 20 seeds. 0, no germination with this treatment. Temperatures, dry after-ripening, and light treatments are statistically significant (*p* < 0.001 by GLM) in these two species. *Post hoc* pairwise *t*-test comparisons (with Bonferroni adjustment) were carried out for each treatment. Bars with different lowercase letters differ significantly (*p* < 0.05) for germination percentage of fresh seeds, and those with different uppercase letters differ significantly (*p* < 0.05) for germination percentage of dry after-ripening seeds at different temperatures. ^∗∗∗^ Indicates significant differences (*p* < 0.001) between germination percentages at the same temperature for the two treatments. The unmarked bars showed no significant difference for each treatment.

In 2014, fresh seeds of *P. sikkimensis* had a relatively high final germination percentage (>70%) at both 25/15 and 15/5°C, whereas *P. secundiflora* seeds had very low germination percentages (<50%), especially under the lower temperature regime (15/5°C, <5.0%; Figure 1). Dry after-ripening for 6 months significantly (*p* < 0.001) increased germination percentages under both temperature regimes in light for these two species (Figure 1).

In 2015, fresh seeds of *P. sikkimensis* germinated well (>90%) at the warmer temperatures (20 and 25°C) in light, but germination significantly decreased to approximately 20.0% at temperatures lower than 20°C (i.e., at 5, 10, and 15°C; Figure 1). Fresh seeds of *P. sikkimensis* incubated in dark also germinated well (88%) at 25°C, but few seeds germinated under the other temperature regimes (5 and 15°C; Figure 1). Fresh seeds of *P. secundiflora* showed a similar trend in light, with seeds germinating at the highest percentages (>80%) at 25°C, and germination percentages significantly decreased to approximately 50.0% at 20°C, and down to 0% at 10 and 5°C (Figure 1). No fresh seeds of *P. secundiflora* germinated in the dark under any temperature regime (Figure 1). The dry after-ripening treatment significantly (*p* < 0.001) increased seed germination percentages and widened the range of germination temperatures in light in both of the species (Figure 1). The effect of dry after-ripening was positive and statistically significant (*p* < 0.001) in dark in *P. sikkimensis*, but not in *P. secundiflora*.

### Germination Tests After Dry Cold Storage

**Figure 2:**
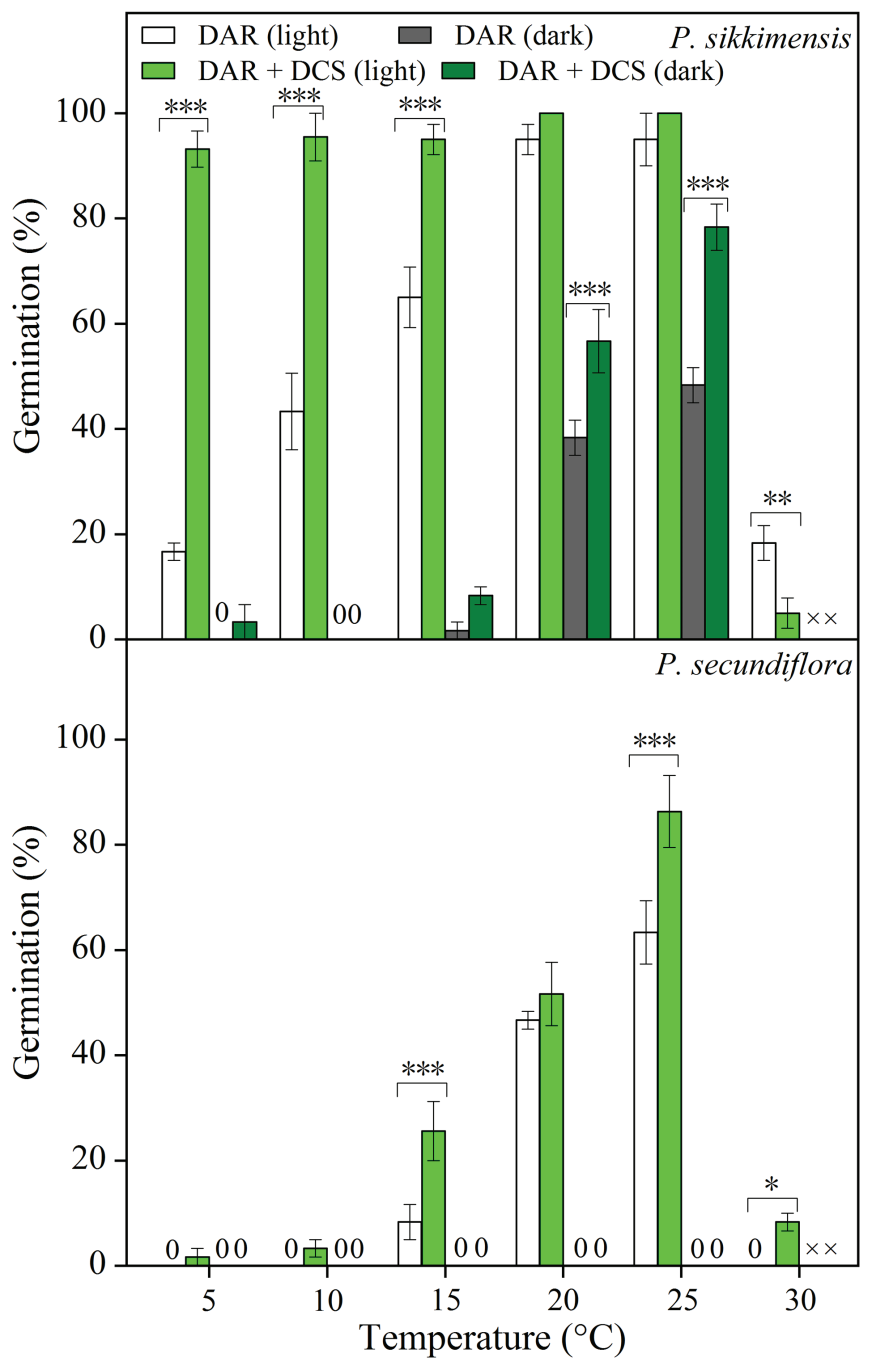
Effects of temperature and dry cold storage treatments (DAR: dry after-ripened at room temperature for 6 months; DAR + DCS: dry cold storage at 4°C after DAR for 4 years) on germination of *Primula secundiflora* and *Primula sikkimensis*. Error bars indicate SE for three replicates of 20 seeds. Temperatures, dry cold storage, and light treatments are statistically significant (*p* < 0.001 by GLM) in these two species. ×, no test in this treatment; 0, no germination with this treatment. *Post hoc* pairwise *t*-test comparisons (with Bonferroni adjustment) were carried out for each germination temperature. ^∗^*p* < 0.05; ^∗∗^*p* < 0.01; and ^∗∗∗^*p* < 0.001, respectively. The unmarked bars showed no significant difference at each temperature.

Four years dry cold storage improved germination significantly, and the fitted GLM highlighted a statistically significant effect (*p* < 0.001) on germination of dry cold storage, temperature, and light condition factors (Figure 2) in both of the species. In *P. sikkimensis*, dry cold storage had a positive and statistically significant effect at lower temperatures (5–15°C) in light, but germination was only improved at higher temperatures (20–25°C) in dark (Figure 2). In *P. secundiflora*, dry cold storage increased germination at lower (15°C) and at higher temperatures (25–30°C; Figure 2), but did not reduce the light requirement of seeds.

### Germination Tests After Cold-Wet Stratification

**Figure 3:**
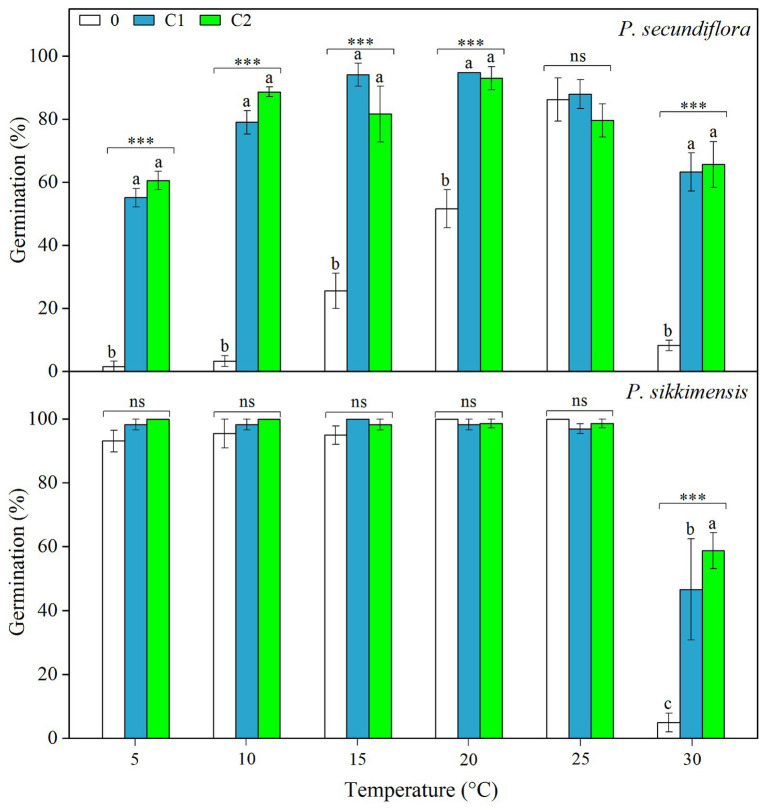
Effects of temperatures and cold treatments (0, control; C1 and C2 cold stratification at 1°C in light for 4 and 8 weeks, respectively) on germination of *Primula secundiflora* and *Primula sikkimensis*. Error bars indicate SE for three replicates of 20 seeds. Temperatures, treatments, and their interaction are statistically significant (*p* < 0.001 by GLM) in these two species. *Post hoc* pairwise t-test comparisons (with Bonferroni adjustment) were carried out for each germination temperature. ^∗∗∗^ Indicates a statistically significant difference at *p* < 0.001, while ns indicates the lack of significant differences between germination percentage at the same temperature for the three treatments. Different letters indicate significant differences at *p* < 0.05 at each temperature.

**Figure 4:**
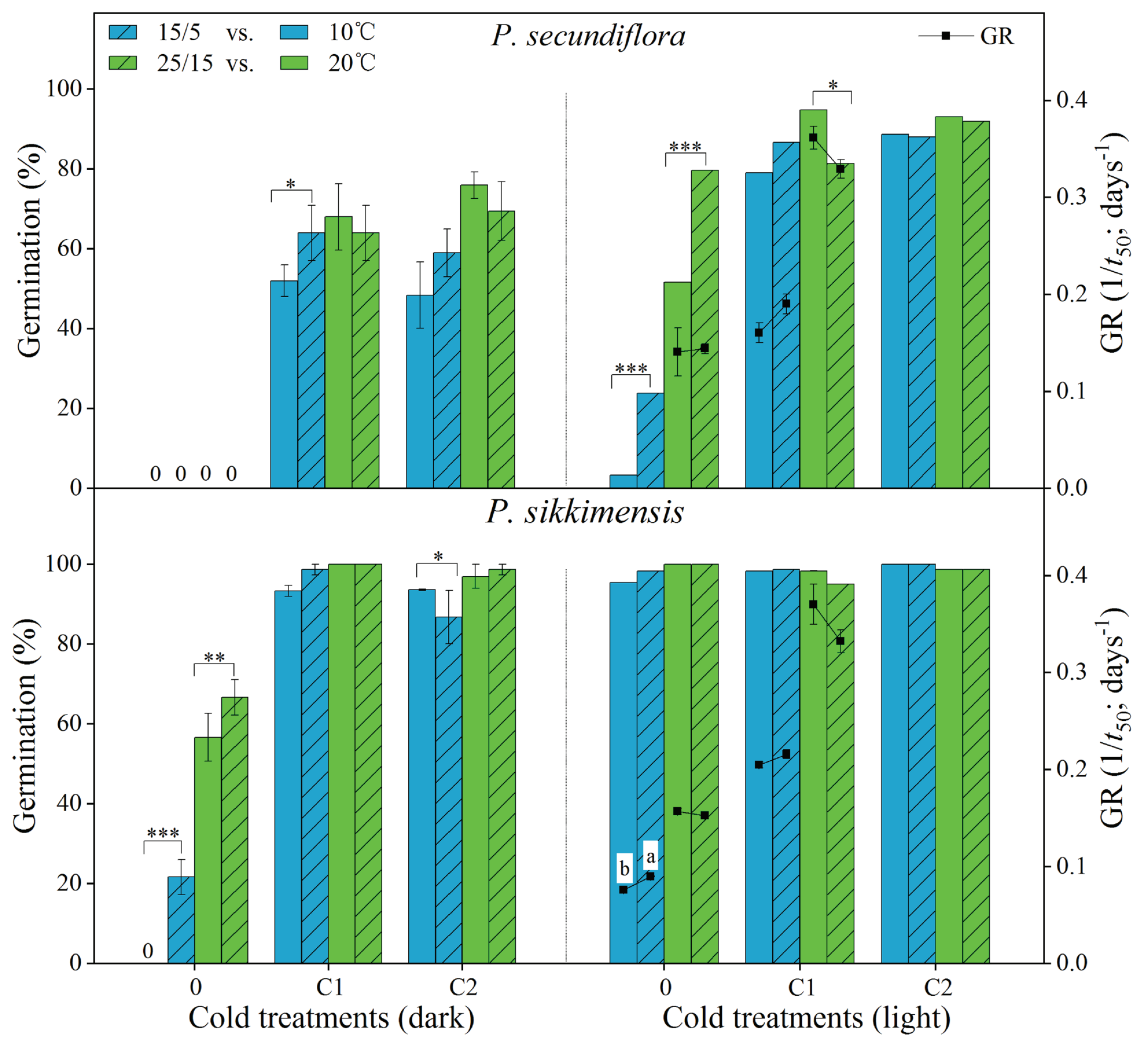
Effect of alternating and corresponding constant temperatures (15/5 vs. 10 and 25/15 vs. 20°C) on final germination percentage and germination rate (GR, 1/*t*_50_) of *Primula secundiflora* and *Primula sikkimensis* under each cold treatment (0, control; C1 and C2 cold stratification at 1°C in light for 4 and 8 weeks, respectively). Error bars indicate SE for three replicates of 20 seeds. 0, no germination with this treatment. ^∗^*p* < 0.05; ^∗∗^*p* < 0.01; and ^∗∗∗^*p* <0.001, respectively. Different letters indicate significant differences in germination rate at *p* < 0.05, and the unmarked bars and boxes show no difference between alternating and corresponding constant temperatures.

Cold stratification treatments increased germination percentage and/or germination rate in light conditions, and the fitted GLM highlighted a statistically significant effect (*p* < 0.001) on germination of temperature and cold-stratified treatment factors and their interaction (Figure 3) in both of the species. In *P. secundiflora*, the germination percentage of control seeds (0) was high (>80%) at higher temperature regimes (25 and 28°C); but below 20 or above 30°C, germination percentage was significantly decreased (Figure 3). The effect of cold stratification was positive and statistically significant at lower temperatures (5, 10, 15, and 20°C) and the higher temperature (30°C; Figure 3); however, germination percentage did not increase in response to the length of stratification (i.e., from 4- to 8-week; Figure 3). In *P. sikkimensis*, the effect of cold stratification was positive and statistically significant at the higher temperature (30°C; Figure 3). Cold stratification also increased germination rate (1/*t*_50_) compared with control seeds at other lower temperatures (10 and 20°C), where there was already high germination (Figure 4).

Our results indicated that, overall, cold stratification, light, and lower alternating temperatures (15/5 vs. 10°C) had significant positive effect on germination in both species (Table 2; Figure 4). In *P. secundiflora*, alternating temperatures regimes (15/5 and 25/15°C) significantly increased germination of control seeds; in contrast, after cold stratification, germination did not increase significantly compared with the corresponding constant temperature in light (10 and 20°C; Figure 4), whereas, cold-stratified seeds (C1) showed a significant germination response to lower alternating temperatures (15/5 vs. 10°C) in dark conditions, but not to higher alternating temperatures (25/15 vs. 20°C; Figure 4). The fitted GLM highlighted a statistically significant effect (*p* < 0.001) on germination of temperature (15/5 vs. 10°C), light and cold stratification treatment factors and their interaction (Figure 4). In *P. sikkimensis*, control and cold-stratified seeds achieved high germination (>95%), regardless of alternating temperature in light (Figure 4), whereas, control seeds showed a significant germination response to alternating temperatures (15/5 vs. 10 and 25/15 vs. 20°C), but not in cold-stratified seeds in the dark (Figure 4). The fitted GLM highlighted a statistically significant effect (*p* < 0.05) on germination of temperature (15/5 vs. 10°C), light and cold stratification treatment factors and their interaction (Table 2; Figure 4).

**Table 2:** GLM results for the effect on seed germination of alternating and corresponding constant temperatures (15/5 vs. 10 and 25/15 vs. 20°C) in relation to the following factors: temperature, light, and cold stratification.

	*Primula secundiflora*				*Primula sikkimensis*			
	10 vs. 15/5°C		20 vs. 25/15°C		10 vs. 15/5°C		20 vs. 25/15°C	
	χ^2^	*P*	χ^2^	*P*	χ^2^	*P*	χ^2^	*P*
Temperature (T)	**12.70**	**0.000**	0.05	0.828	**8.60**	**0.003**	0.78	0.376
Light (L)	**108.31**	**0.000**	**252.97**	**0.000**	**607.95**	**0.000**	**93.67**	**0.000**
Cold stratification (C)	**663.13**	**0.000**	**390.42**	**0.000**	**901.83**	**0.000**	**165.43**	**0.000**
T^∗^L	0.12	0.733	3.12	0.077	**4.53**	**0.033**	3.06	0.080
T^∗^C	1.05	0.592	**18.72**	**0.000**	**23.50**	**0.000**	4.98	0.083
L^∗^C	**13.47**	**0.001**	**87.49**	**0.000**	**807.66**	**0.000**	**215.37**	**0.000**
T^∗^L^∗^C	**8.54**	**0.014**	**11.66**	**0.003**	**15.48**	**0.000**	2.71	0.258

Significant effects (*p* < 0.05) are highlighted in bold.

#### Thermal Requirement for Germination

**Figure 5:**
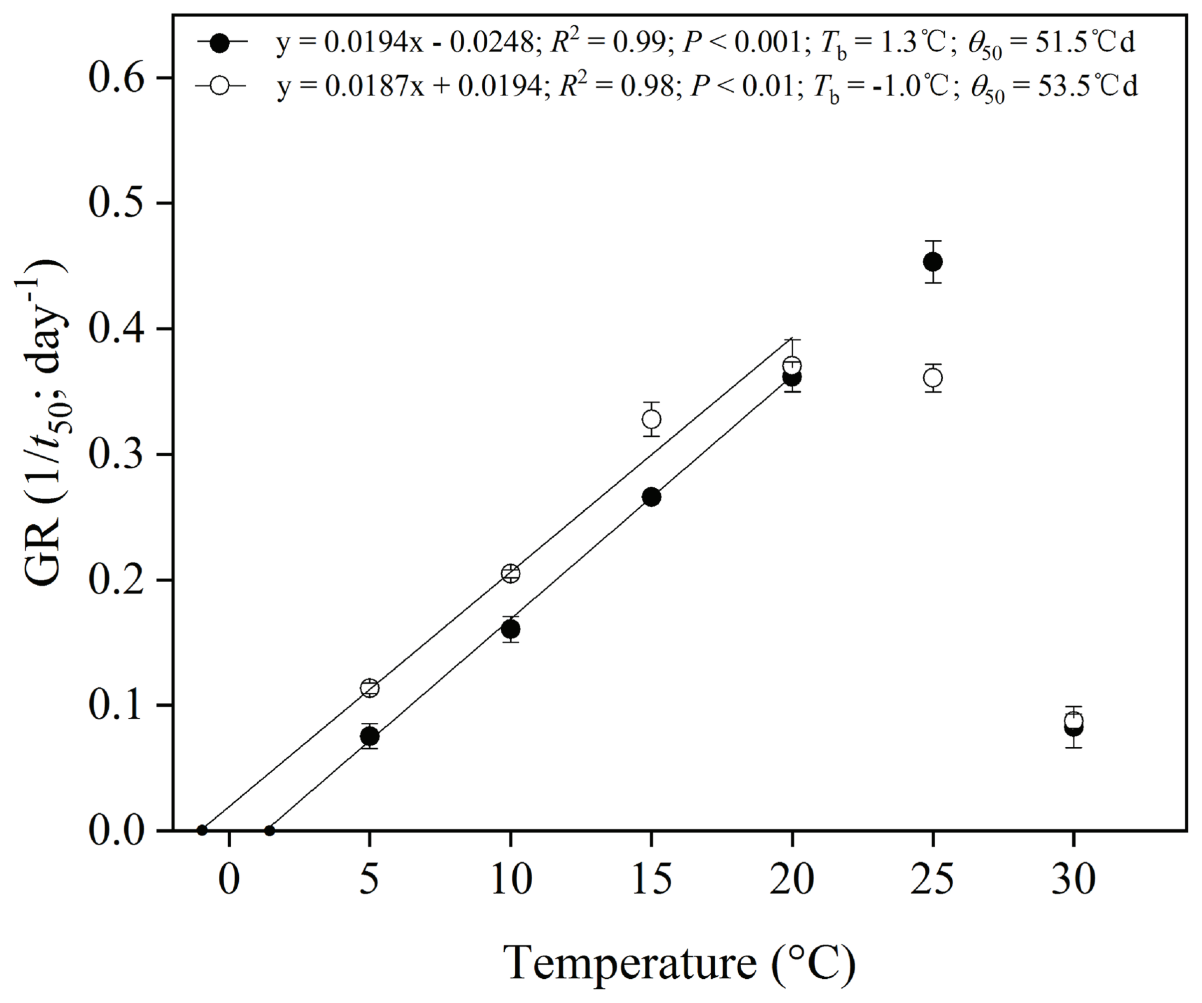
Base temperatures (*T*_b_) and thermal times calculated for 50% germination percentiles of *Primula secundiflora* (solid circle) and *Primula sikkimensis* (empty circle) seeds, after cold stratification at 1°C in light for 4 weeks and incubation at 5–30°C (the sub-optimal temperatures: 5–25°C). Error bars indicate SE for three replicates of 20 seeds. Points correspond to the actual data and lines indicate the fitted line from the linear regressions.

The reciprocal of the time taken to reach 50% germination, the 1/*t*_50_, was plotted against temperature to establish a thermal time model for cold-stratified seeds for 4 weeks (Figure 5). Goodness of fit (*R*^2^) for the linear regressions of 1/*t*_50_ against temperature showed that the best sub-optimal model included data only up to 20°C (i.e., excluding 25–30°C; Figure 5). It was possible to estimate the base temperature for germination (*T*_b_) in the suboptimal temperature range (Figure 5). *T*_b_ values and thermal time (*θ*_50_) were 1.3 and −1.0°C, 51.5 and 53.5°Cd for *P. secundiflora* and *P. sikkimensis*, respectively.

### Soil Seed Bank

**Figure 6:**
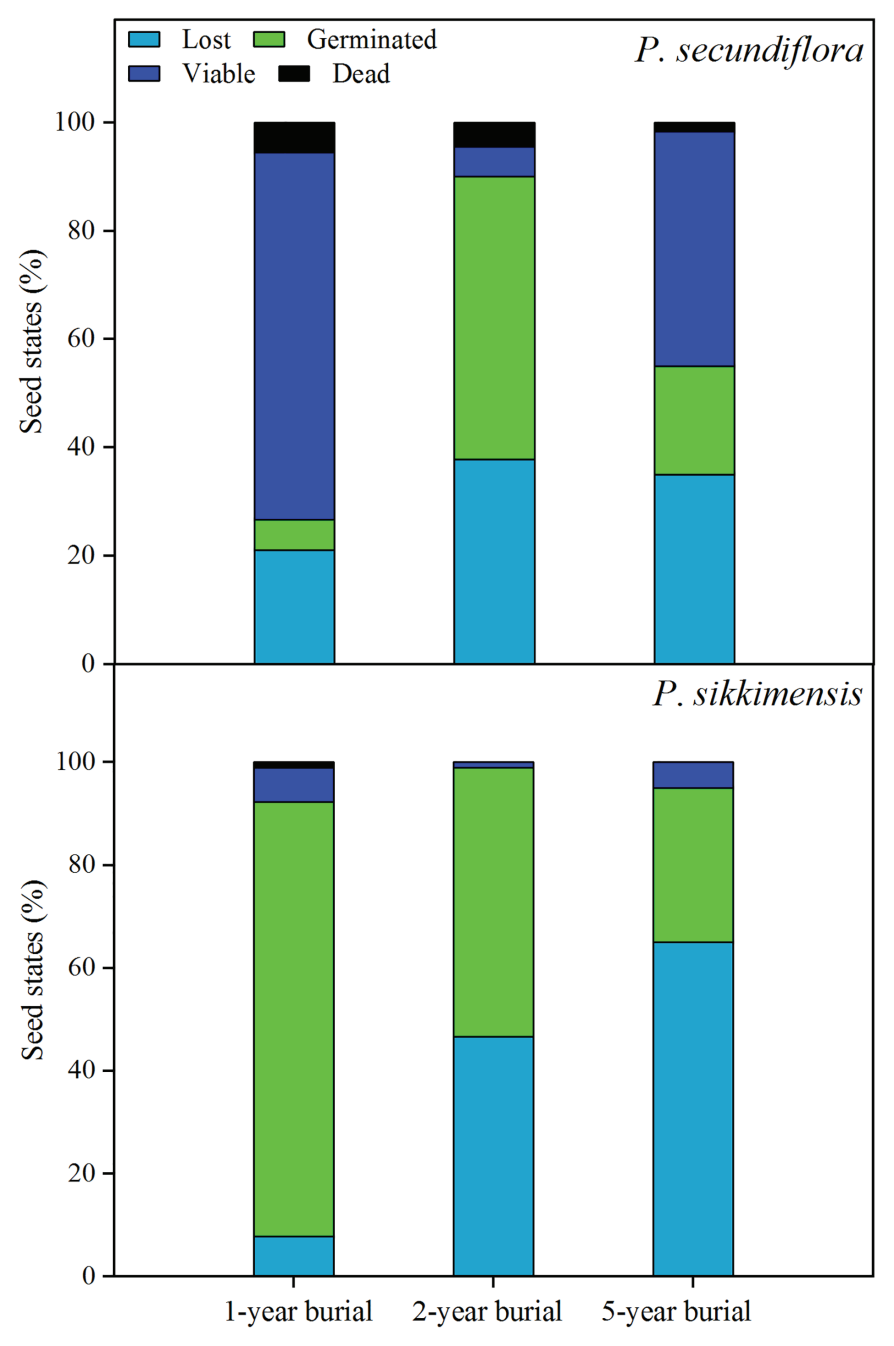
The fraction of lost, germinated, viable, and dead seeds of *Primula secundiflora* and *Primula sikkimensis* in the 1st-, 2nd-, and 5th-year after buried. ***Lost***, seed germinated and/or died in the soil whist buried; ***Germinated***, seed germinated after retrieved; ***Viable***, seed did not germinate but could still be stained; ***Dead***, the remaining proportion. The proportion of seeds in each category was calculated for 30 seeds initially put in the replicate seed bags.

At the end of the 5-week germination period on the water agar substrate before seed burial in 2014, many seeds had already germinated (Figure 1) and the majority of the ungerminated seeds were still viable (total seed viability for *P. secundiflora*: 100.0 ± 0%; *P. sikkimensis*: 98.6 ± 1.5%). One year after seed burial, 78.9% of the *P. secundiflora* seeds, and 92.2% of the *P. sikkimensis* seeds could be retrieved, indicating that a low percentage of seeds was loss due to seed germination and/or attacked by insects or fungi in the soil during winter and the first growing season (Figure 6). Seed bank lost seemed to be high as only 46.7% of the *P. sikkimensis* seeds and 62.0% of the *P. secundiflora* seeds could be retrieved after 2-year (Figure 6). The majority of the retrieved *P. secundiflora* seeds delayed germination for 2-year, while the fraction of *P. sikkimensis* seeds germinating decreased as seeds aged (Figure 6). Nevertheless, a subset of seeds was viable even after having been buried for 5-year (*P. secundiflora*: 63%; *P. sikkimensis*: 35%).

## Discussion

Our study demonstrated similar patterns of seed dormancy and germination responsiveness to temperature and light after dormancy release in two alpine *Primula* species. Additionally, we provided direct field evidence indicating that the light requirements for germination influence seed germination in the soil, likely contributing to a persistent soil seed bank. Taken together, such dormancy mechanism, the light and temperature requirements may delay germination until favorable time and place, representing an advantageous ecological adaptation toward the unpredictable alpine environments.

### Type of Dormancy

In this study, we found evidence that both *P. secundiflora* and *P. sikkimensis* seeds exhibit type 3 non-deep PD. The dormancy type of these two species was demonstrated in three ways. Freshly-matured seeds had fully developed embryos, and germination was significantly improved by dry after-ripening at room temperature (Figure 1). These results suggested that fresh seeds may exhibit have PD and supported earlier observations of other *Primula* species (Hitchmough et al., 2011; Wang et al., 2017; Peng et al., 2019a; Yang et al., 2020). Second, dry after-ripening was not sufficient to break dormancy, but then GA_3_ and dry cold-storage could still significantly increase germination percentage (Figure 2; Supplementary Figure S1), indicating that seeds of the two *Primula* species appear to have non-deep PD (Baskin and Baskin, 2014, 2020). Further, cold stratification and dry cold-storage had positive effects on germination (Figures 2, 3), and the widening of the temperature range at which seeds reached high germination percentages − from medium to both high and low – indicated that both *P. secundiflora* and *P. sikkimensis* seeds exhibit type 3 non-deep PD (Baskin and Baskin, 2014). Such a dormancy mechanism prevents germination immediately after seed dispersal in the autumn because the temperature required for germination is high, and this temperature is not reached in alpine environments during autumn.

### Thermal Requirement for Germination After Dormancy Release

After overwintering, cold stratification reduces the high temperature requirement and modifies the cardinal temperatures (e.g., *T*_b_) for seeds belonging to the same seed lots (Orrù et al., 2012). The base temperatures for germination (*T*_b_) in seeds of *P. secundiflora* varied from approximately 10°C for control seeds (0) to 1.3°C for cold-stratified seeds (4 weeks), whereas seeds of *P. sikkimensis* had a lower *T*_b_, its value was as low as −1.0°C (Figure 4). Seeds of the two *Primula* species had similar low *T*_b_ values (<5°C) as other *Primula* species in the alpine region of the Hengduan Mountains (Peng et al., 2019a; Yang et al., 2020). The detected *T*_b_ values are consistent with the current altitude (>4,500 m a.s.l.) of these two species. Such an environment, seed dormancy, and thermal characteristics of these species would probably prevent seed germination after seed dispersal in higher elevation regions, where autumn and winter temperatures are too low to facilitate seed germination. The requirement of cold stratification for release from dormancy is a universal mechanism for preventing winter germination and is shared by most spring-germinating alpine plants (Mondoni et al., 2009; Schwienbacher et al., 2011; Cavieres and Sierra-Almeida, 2018; Yang et al., 2020).

### Light Requirement for Germination Affecting Seed Persistence

Seeds requiring light for germination are usually small in size (Milberg et al., 2000). In our study, both of the *Primula* species exhibited a light requirement for germination before cold stratification (Table 2; Figures 2, 4). These results are in agreement with those of previous studies on other *Primula* species with relatively small seeds (Shimono and Washitani, 2004; Hitchmough et al., 2011; Wang et al., 2017; Peng et al., 2019a; Yang et al., 2020). However, *P. secundiflora* seeds had a more strict light requirement for germination than seeds of *P. sikkimensis*, and the latter had a relatively high germination percentage at the intermediate constant temperatures (20–25°C) in the dark (Figures 1, 2, 4). Seed weight and habitat preference may be the cause of the different light requirements for germination. The light requirement for germination was stronger in smaller than in larger seeded species that were closely related (Koutsovoulou et al., 2014). In these two primrose species, *P. secundiflora* has relatively small seeds (Table 1), which might result in a high light requirement for germination; however, a relatively high germination percentage in the dark in *P. sikkimensis* cannot be ascribed unequivocally to seed weight since it may also be an effect of the drier habitat. *Primula secundiflora* seeds were collected alongside a stream in an alpine meadow, and this habitat is generally wet in spring; therefore, this requirement for light, which is a common germination requirement or germination stimulus (Schütz and Rave, 1999), may be an adaptation to wet habitats. *Primula sikkimensis* occupied somewhat drier habitats of meadow patches in alpine scree, which might also result in a relatively low light requirement for germination.

Fresh seeds of the two *Primula* species may have failed to germinate due to dormancy mechanisms (PD), and then these small seeds were easily buried deep in the soil. Our data indicated that, at least for dormant seeds, the light requirement for germination (Figures 1, 2, 4) explained their inability to germinate in the soil, resulting in seed present in the soil after at least 5 years (Figure 6). According to the soil seed bank classification of Peco et al. (2003), both *Primula* species produced a long-term persistent soil seed bank. The light requirement for germination may be reduced during treatments for dormancy release (Milberg and Noronha, 1996; Schütz, 1997). In our study, the dormancy-breaking treatments (e.g., dry storage and cold stratification) not only broke dormancy at low temperatures, but also significantly reduced the light requirement for germination (Figures 2, 4). These results support earlier observations of other *Primula* species (Milberg, 1994; Hitchmough et al., 2011; Wang et al., 2017). The light requirement may also change in response to daily temperature fluctuation in many species (Baskin and Baskin, 2014). By comparing germination between alternating (15/5°C) and constant (10°C) temperature regimes, we showed that the light requirement is partially eliminated by lower alternating temperatures (15/5°C; Table 2; Figure 4), and this temperature regime can occur in alpine regions near the surface of the soil during the germination period of most spring-germinating species (end of April to beginning of May). These mechanisms ensure that germination occurs only in gaps and at/near the soil surface, thus avoiding seedling death by inundation or germination deep in the soil. If viable seeds in the soil seed bank do not germinate in spring they can re-enter dormancy, because of temperature-driven cycling of dormancy in seeds exhibiting non-deep PD (Wang et al., 2017). Such dormancy cycling, a germination response to fluctuating temperatures and light, allows these two species to germinate at the right time (immediately after snowmelt) of the year, enabling plants to cope with harsh and unpredictable alpine environmental conditions (Schwienbacher et al., 2010).

## Conclusion

In conclusion, type 3 non-deep PD was identified for fresh seeds of the two *Primula* species. After dormancy release, *T*_b_ and *θ*_50_ for germination decreased. Such dormancy mechanisms and thermal characteristics for germination suggest that seeds may delay germination until the next spring. The light requirement for the germination of the two *Primula* species explained their ability to produce a long-term persistent soil seed bank; however, the light requirement was reduced during treatments for dormancy release or at lower alternating temperatures (15/5°C). These mechanisms for germination allow buried seeds to sense depth and may initiate germination of seeds within suitable gaps when water levels, light, and temperatures are suitable, which may be advantageous in alpine habitats (Peng et al., 2017, 2018, 2019b).

## Data Availability Statement

The original contributions presented in the study are included in the article/Supplementary Material, further inquiries can be directed to the corresponding authors.

## Author Contributions

D-LP and Z-ML designed the study. L-EY, D-LP, and JY performed the experiments. D-LP analyzed the data. D-LP and L-EY wrote the first draft of the manuscript. All authors contributed to the article and approved the submitted version.

### Conflict of Interest

The authors declare that the research was conducted in the absence of any commercial or financial relationships that could be construed as a potential conflict of interest.
