# Supplementary material for: Seed Dormancy and Soil Seed Bank of the Two Alpine Primula Species in the Hengduan Mountains of Southwest China
Source: Front Plant Sci. 2021 Apr 14;12:582536. doi: 10.3389/fpls.2021.582536 (PMC8081389; doi:10.3389/fpls.2021.582536)
Supplement: Supplementary file 1 [file Data_Sheet_1.DOCX]

**Seed germination experiments**

**Materials and methods**

To determine the level of physiological dormancy after seed (using seeds collected in 2013) dry after-ripening at room temperature for six months and dry cold storage at 4 °C for five years, and then seeds were incubated on 1% water agar substrate with 100 mg∙L^-1^ of GA_3_ at the two alternating temperatures (15/5, 25/15 °C). Three replicates of 20 seeds were used in each treatment. The Petri dishes were put into transparent plastic bags to prevent desiccation. Seeds incubated in the light were counted daily and germinated seeds were discarded.

**Data analysis**

We applied an independent sample t-test to determine whether GA_3_ had significantly increased seed germination after dry after-ripening and dry cold storage.

**Results**

For seeds that dry after-ripened for 6 months, GA_3_ increased final germination percentage significantly at 25/15 and 15/5 °C in both *Primula secundiflora* and *P. sikkimensis* (Figure S1). After seeds dry cold storage for five years, final germination percentage was still significantly increased in GA_3_-treated than control seeds at 15/5°C in *P. secundiflora*, but not increased significantly at other treatments in these two species (Figure S1).





Figure S1. Effect of GA_3_ on final germination percentages of seeds after dry after-ripening and cold storage in *Primula secundiflora* and *P. sikkimensis* at 25/15 and 15/5 °C. Error bars indicate SE for three replicates of 20 seeds. *** indicate significant difference at 0.001 levels, and the unmarked bars showed no difference between GA_3_-treated and control (Ctrl) seeds.
